# Crossmodal Congruency Enhances Performance of Healthy Older Adults in Visual-Tactile Pattern Matching

**DOI:** 10.3389/fnagi.2020.00074

**Published:** 2020-03-17

**Authors:** Focko L. Higgen, Charlotte Heine, Lutz Krawinkel, Florian Göschl, Andreas K. Engel, Friedhelm C. Hummel, Gui Xue, Christian Gerloff

**Affiliations:** ^1^Department of Neurology, University Medical Center Hamburg-Eppendorf, Hamburg, Germany; ^2^Department of Neurophysiology and Pathophysiology, University Medical Center Hamburg-Eppendorf, Hamburg, Germany; ^3^Defitech Chair of Clinical Neuroengineering, Brain Mind Institute and Center for Neuroprosthetics, Swiss Federal Institute of Technology (EPFL), Geneva, Switzerland; ^4^Defitech Chair of Clinical Neuroengineering, Brain Mind Institute and Center for Neuroprosthetics, Swiss Federal Institute of Technology Valais (EPFL Valais), Clinique Romande de Réadaptation, Sion, Switzerland; ^5^Clinical Neuroscience, Medical School University of Geneva, Geneva, Switzerland; ^6^State Key Laboratory of Cognitive Neuroscience and Learning, Beijing Normal University, Beijing, China

**Keywords:** aging, elderly, integration, multisensory, rehabilitation

## Abstract

One of the pivotal challenges of aging is to maintain independence in the activities of daily life. In order to adapt to changes in the environment, it is crucial to continuously process and accurately combine simultaneous input from different sensory systems, i.e., crossmodal or multisensory integration. With aging, performance decreases in multiple domains, affecting bottom-up sensory processing as well as top-down control. However, whether this decline leads to impairments in crossmodal interactions remains an unresolved question. While some researchers propose that crossmodal interactions degrade with age, others suggest that they are conserved or even gain compensatory importance. To address this question, we compared the behavioral performance of older and young participants in a well-established crossmodal matching task, requiring the evaluation of congruency in simultaneously presented visual and tactile patterns. Older participants performed significantly worse than young controls in the crossmodal task when being stimulated at their individual unimodal visual and tactile perception thresholds. Performance increased with adjustment of stimulus intensities. This improvement was driven by better detection of congruent stimulus pairs, while the detection of incongruent pairs was not significantly enhanced. These results indicate that age-related impairments lead to poor performance in complex crossmodal scenarios and demanding cognitive tasks. Crossmodal congruency effects attenuate the difficulties of older adults in visuotactile pattern matching and might be an important factor to drive the benefits of older adults demonstrated in various crossmodal integration scenarios. Congruency effects might, therefore, be used to develop strategies for cognitive training and neurological rehabilitation.

## Introduction

As the percentage of older people in the population increases, aging-related declines gain more and more significance. An important endeavor, therefore, is to identify means for supporting older adults to maintain sound minds and independent living.

In order to behave adequately in our natural environment, it is crucial to continuously process simultaneous input from different sensory systems and integrate this information into meaningful percepts (Meredith and Stein, 1983, 1986; Calvert, 2001; Spence, 2007). This crossmodal or multisensory integration (for definition see Calvert, 2001) complements unimodal sensory perception and allows for basing decisions and behavior on a broader range of sensory cues (Calvert et al., 2004). However, the relevance of crossmodal integration in older adults is still under debate (for example see Cienkowski and Carney, 2002; Setti et al., 2011; Freiherr et al., 2013; McGovern et al., 2014). While some authors report that the neurocomputational integration of multiple sensory stimuli degrades with age (e.g., Stine et al., 1990; Sommers et al., 2005; Stephen et al., 2010), others suggest that crossmodal integration is conserved or even gains compensatory importance in older adults (e.g., Laurienti et al., 2006; Peiffer et al., 2007; Diederich et al., 2008; Diaconescu et al., 2013).

Age-related decline affects processes of crossmodal interactions in several ways. The bottom-up processing of sensory stimuli constitutes one of the key features of this deterioration. Age-related sensory impairments affect all modalities. This is mirrored in decreased acuity in visual, auditory or tactile detection tasks (Kenshalo, 1986; Kalina, 1997; Jackson and Owsley, 2003; Poliakoff et al., 2006a; Wickremaratchi and Llewelyn, 2006; Davis et al., 2016) as well as increased thresholds for taste and odor detection (Schiffman, 1997; Spence, 2012). Adding to the decline of peripheral sensory organs, aging also affects cognitive domains highly relevant to the top-down control of crossmodal interactions. Older adults show for example deficits in attention, divided attention, working memory, episodic memory and decision making (Gazzaley et al., 2005; Anguera and Gazzaley, 2012; Fraser and Bherer, 2013; Guerreiro et al., 2014).

Alterations in both, bottom-up stimulus processing as well as top-down control suggest that crossmodal interactions should decrease with age. This is line with classical studies postulating that the decline in sensory organs and higher cognitive domains prevent older adults from taking advantage of crossmodal information, by restricting effective multisensory integration processes and limiting the cognitive resources needed (e.g., Stine et al., 1990).

However, there is accumulating evidence that points to enhanced crossmodal interactions in older adults (e.g., Laurienti et al., 2006; Peiffer et al., 2007; Diederich et al., 2008; Diaconescu et al., 2013). Different age-related alterations in central neurocomputational processes have been discussed as possible reasons for this enhancement (for review, see Mozolic et al., 2012; Freiherr et al., 2013). One potential reason that has been suggested is the decline in the unimodal sensory stimulus processing described above (Hairston et al., 2003; Freiherr et al., 2013). According to a classic principle of multisensory integration called inverse effectiveness, decreasing the effectiveness of individual sensory stimuli increases the magnitude of multisensory enhancements (Meredith and Stein, 1983, 1986; Holmes and Spence, 2005). Apart from that, general cognitive slowing in older adults, demonstrated in several tasks (Cerella, 1985; Birren and Fisher, 1995; Salthouse, 2000), has been suggested to lead to more susceptibility to crossmodal integration by extending the temporal window for possible cross-modal interactions (Verhaeghen and De Meersman, 1998; Setti et al., 2014). Furthermore, it has been proposed that gains in performance in scenarios with crossmodal stimulation (Hugenschmidt et al., 2009a; Mozolic et al., 2012) might relate to increases in baseline crossmodal interactions in older adults due to neural noise (Hugenschmidt et al., 2009b; Voytek and Knight, 2015). A functional consequence common to the proposed age-related alterations in central processing is that they should lead to enhanced crossmodal interactions in various crossmodal stimulation scenarios, even in scenarios where multisensory integration is not facilitated in a bottom-up manner. However, it is not clear how this applies to scenarios affected by the above-described decline of top-down mechanisms with aging, such as divided attention.

To further evaluate whether aging leads to enhanced crossmodal interactions, we investigated group differences between healthy older and younger participants in a well-established visuotactile matching task (Hummel and Gerloff, 2006; Göschl et al., 2014, 2015; Wang et al., 2019). In this task, participants have to evaluate congruency in simultaneously presented visual and tactile dot patterns. Most studies that found a behavioral benefit of older adults in crossmodal tasks have focused on visual-auditory integration (e.g., Laurienti et al., 2006; Peiffer et al., 2007; Diederich et al., 2008). Data on visuotactile interactions in older adults are sparse (Poliakoff et al., 2006a,b; Lee et al., 2009). However, the sense of touch has been shown to be immensely important in all areas of everyday life (Gallace and Spence, 2014). Furthermore, there is evidence suggesting that tasks involving the interaction of visual and somatosensory stimuli profit strongly from crossmodal interaction effects (Mahoney et al., 2011; Misselhorn et al., 2016). The tactile modality interacts with vision for example in object recognition or the identification of somatosensory stimuli but also in posture control (Tipper et al., 1998; Oie et al., 2002; List et al., 2012; Gallace and Spence, 2014). These represent basic abilities needed for interacting with the environment and to preserve independence.

To be able to compare participants’ crossmodal performance and the subjective task difficulty across both groups and modalities, we determined individual unimodal perception thresholds prior to the crossmodal experiment (Beer and Röder, 2004; Poole et al., 2015; Venkatesan et al., 2018). Using the individual unimodal perception thresholds in the visuotactile matching task allowed us to assess differences in crossmodal performance between the two groups not related to unimodal stimulus processing, but to crossmodal task demands.

Our first hypothesis was that unimodal perception thresholds for visual and tactile pattern recognition should be higher in the older group compared to younger, due to multiple age-related sensory impairments (Mancini and Allen, 2018). Second, we hypothesized that older participants would show enhanced crossmodal interactions compared to younger in the visuotactile matching task involving stimuli presented at the individual unimodal perceptual thresholds—in accordance with the proposed mechanisms of enhanced crossmodal interactions with aging described above.

## Materials and Methods

### Participants

Thirty-seven older and 22 younger volunteers were screened for the study. Six older volunteers did not meet the inclusion criteria during the initial assessment. One older and two younger participants dropped out because of personal reasons or technical problems. Ten older participants (five females, mean (M) = 74.1 years, standard deviation (SD) = 3.90 years) did not meet the predefined accuracy criterion in a training session prior to the threshold estimation (described in detail in “Experimental Procedure” section) and were no longer considered in the analysis. Thus, the final sample consisted of 20 younger (11 females, *M* = 24.05 years, *SD* = 2.50) and 20 older (11 females, *M* = 72.14 years, *SD* = 4.48) volunteers. Assuming normality of the data distribution, an *a priori* sample size calculation was conducted based on a power calculation for a repeated-measures ANOVA (with within-between interaction) for two groups and three measurements (habituation task, unimodal training, visuotactile matching) with a statistical power of 90% and a type-1 error of 5% (effect size 0.25), which results in a total sample size across two groups of 36. All participants were right-handed according to the Edinburgh handedness inventory (Oldfield, 1971), had normal or corrected to normal vision, no history or symptoms of neuropsychiatric disorders (MMSE ≥ 28, DemTect ≥ 13) and no history of centrally acting drug intake. All participants received monetary compensation for participation in the study.

### Statement of Ethics

The study was conducted in accordance with the Declaration of Helsinki and was approved by the local ethics committee of the Medical Association of Hamburg (PV5085). All participants gave written informed consent.

### Assessment

Prior to inclusion, each participant underwent an assessment procedure. The assessment consisted of a neurological examination, the Mini-Mental State Examination (MMSE; Folstein et al., 1975) and the DemTect (Kalbe et al., 2004) to rule out symptoms of neuropsychiatric disorders. Furthermore, a 2-point-discrimination test (cut off > 3 mm; Crosby and Dellon, 1989; Dellon et al., 1995) and a test of the mechanical detection threshold (cut off > 0.75 mN; MDT, v. Frey Filaments, OptiHair2-Set, Marstock Nervtest, Germany; Fruhstorfer et al., 2001; Rolke et al., 2006) were conducted to ensure intact peripheral somatosensation.

### Setup and Stimuli

The experiment was conducted in preparation for a magnetoencephalography (MEG) study and the setup was designed to match conditions in the MEG laboratory. The experiment took place in a light-attenuated chamber. We chose the experimental procedure, stimulus configuration and stimulation parameters based on pilot data showing the accuracy of tactile pattern recognition to be very different between older and younger participants.

**Figure 1:**
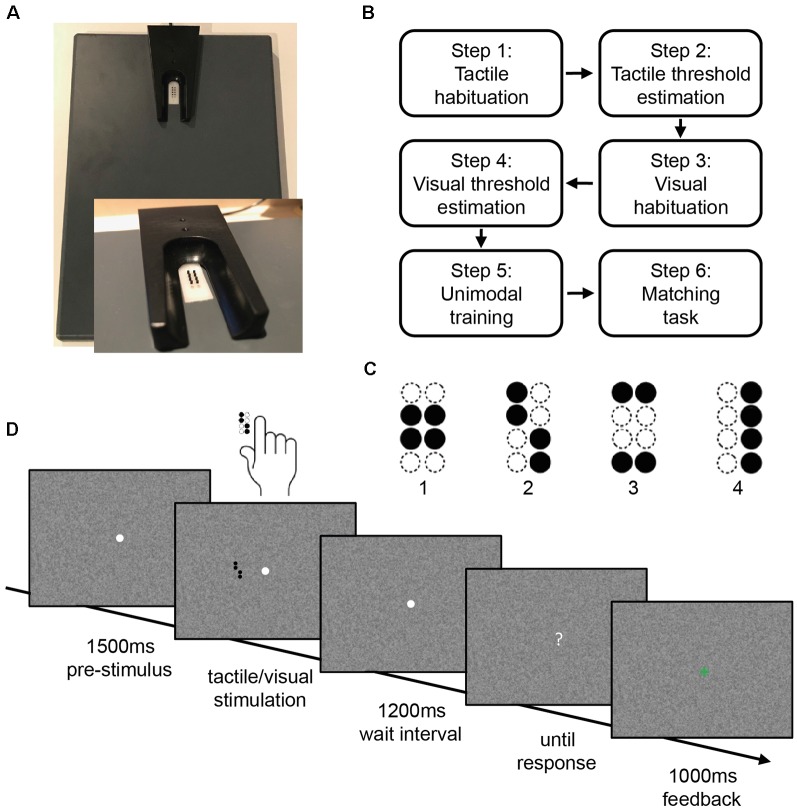
Stimulus design and experimental procedure. **(A)** Braille stimulator. For tactile stimulation, the participants’ right hand was resting on a custom-made board containing the Braille stimulator (QuaeroSys Medical Devices, Schotten, Germany), with the fingertip of the right index finger placed above the stimulating unit. **(B)** Sequence of tasks in the experiment. **(C)** Stimuli consisted of four different patterns. **(D)** After a pre-stimulus interval of 1,500 ms, tactile and/or visual patterns were presented for 500 ms depending on the current step of the experiment. After a wait interval of 1,200 ms, a question mark appeared on the screen and participants gave the response *via* button press. Depending on the current step of the experiment, visual feedback was given (1,000 ms).

We used an adapted version of a well-established experimental paradigm, the visuotactile matching task (Göschl et al., 2014, 2015; Hummel and Gerloff, 2006; Wang et al., 2019). Participants are instructed to compare tactile patterns presented to the right index fingertip and visual patterns presented on a computer screen. For tactile stimulation, the participants’ right hand was resting on a custom-made board containing a Braille stimulator (QuaeroSys Medical Devices, Schotten, Germany, see Figure 1A). The Braille stimulator consists of eight pins arranged in a four-by-two matrix, each 1 mm in diameter with a spacing of 2.5 mm. Each pin is controllable independently. Pins can be elevated for any period of time to form different patterns. At the end of each pattern presentation, all pins return to baseline. The stimuli consisted of four geometric patterns, each of them formed by four elevated pins (Figure 1C). Participants passively perceived the elevated pins without active exploration. A 15-inch screen at 60 Hz with a resolution of 1,024 × 768 pixels positioned 65 cm in front of the participants served for presentation of the visual stimuli. The design of the visual patterns was analogous to the tactile patterns. The visual patterns subtended 3.5° × 2.5° of visual angle. They were presented left of a central fixation point on a noisy background (Perlin noise; Figure 1D).

Depending on the task, the amplitude of pin elevation and the gray intensity of visual patterns were adjusted, while the duration of the pattern presentation was always kept constant at 500 ms. The amplitude of the pin elevation can be controlled in 4,095 discrete steps, with a maximum amplitude of 1.5 mm. Maximal gray intensity equaled black patterns with RGB: 0-0-0.

We used Presentation software (Neurobehavioral Systems, version 15.1) to control stimulus presentation and to record participants’ response time (RT) and accuracies.

### Experimental Procedure

All participants who met the predefined accuracy criterion in a training session prior to the experiment (at least 75% correct answers in a tactile-to-visual delayed match-to-sample task with easy tactile patterns) performed a series of tasks representing the current experiment (tactile habituation, tactile threshold estimation, visual habituation, visual threshold estimation, unimodal training, matching task; see Figure 1B). At the beginning of each task, participants read the task instructions presented on a computer screen. The experiment started with the tactile habituation task.

#### Tactile Habituation

The tactile habituation task consisted of a tactile-to-visual delayed match-to-sample task. Four target patterns were introduced as the stimulus set (Figure 1C), at maximum pin amplitude and with a duration of 500 ms. We decided to use a delayed match-to-sample task for habituation and threshold estimation as for the visuotactile matching task participants had to reliably identify each of four geometric patterns in both modalities to be able to compare them. Furthermore, in the visuotactile matching task, participants had to process each of the two unimodal stimuli, maintain representations of the two patterns and compare them. We designed the delayed match-to-sample task to address these task demands and to be consistent with the trial sequence of the visuotactile matching task (see Figure 1D). Each trial started with a central white fixation point appearing on a noisy background. This fixation point remained visible throughout each trial. The tactile pattern presentation started 1,500 ms after the appearance of the fixation point with a stimulus chosen pseudo-randomly from the stimulus set. After the tactile presentation and a waiting interval of 1,200 ms, the central fixation point turned into a question mark and participants indicated which of the four patterns had been presented. Participants responded *via* button press with the fingers 2–5 of the left hand. After each trial participants received visual feedback (1,000 ms) whether their response was correct (green “+”) or incorrect (red). The waiting interval after stimulus offset was integrated to prepare for the following MEG experiment, where it allowed for avoiding motor artifacts in the MEG signal. The background changed after every trial. After a minimum of five training blocks, each consisting of 16 trials, and an accuracy of at least 75% in three of five consecutive blocks, participants could proceed to the next step. If participants did not reach the target accuracy within 15 blocks, they were excluded from further participation.

#### Tactile Threshold Estimation

Pilot studies indicated that most older adults were able to recognize the target patterns at 500 ms stimulus presentation time in the unimodal tactile condition with an accuracy of approximately 80% correct. However, using the same parameters, younger performed close to 100%. Equally, visual recognition accuracy was close to 100% in both groups for these parameters. To achieve a comparable performance of around 80% correct answers for both modalities in older and younger participants, we conducted an adaptive staircase procedure to detect thresholds for visual and tactile pattern recognition and tailor stimulus intensities for each participant.

Since the slope of the psychometric function was supposed to be very different in older and younger participants and we did not have any priors regarding the exact shape, we decided not to use a Bayesian approach (e.g., Quest; Watson and Pelli, 1983), but to implement a non-parametric adaptive staircase procedure (García-Pérez, 1998; Wetherill and Levitt, 1965; Kaernbach, 1991; Treutwein, 1995). We designed a two-down/one-up fixed-step-size adaptive staircase. With a ratio Δ-down/Δ-up = 0.5488, this staircase should converge around 80.35%. For tactile pattern presentation, an adaptation of the height of the braille pins rendered recognition easier or more complicated. Step size was determined after piloting with approximately 0.1 mm up, 0.055 mm down. The staircase started with a maximum amplitude of 1.5 mm. The staircase stopped after 20 reversals while proceeding at boundary levels. The last 16 reversals served to calculate thresholds. Participants performed this staircase for both unimodal visual and unimodal tactile stimulation. Trial timing was the same as in the habituation task, except there was no feedback given.

#### Visual Habituation

The visual habituation task followed the same procedure as in the tactile condition. Instead of tactile stimulation, patterns were presented visually at maximal contrast (see Figure 1C, target patterns). Again, participants continued to fixate the central point during pattern presentation, so that visual patterns would appear in the left visual hemifield. Trial timing, block design, and accuracy criterion were the same as for the tactile recognition task.

#### Visual Threshold Estimation

The visual threshold estimation followed the same procedure as in the tactile modality. For visual threshold estimation, an adaptation of the gray intensity of the pattern varied the patterns’ contrast against the noisy background. Step size was determined after piloting, with a step up being two intensities, and a step down one on the grayscale ranging from 47 (RGB: 138-138-138) to 101 (RGB: 0-0-0). The staircase started with the maximum contrast (black pattern; RGB: 0-0-0). Pilot data showed that a gray intensity of RGB: 138-138-138, which corresponds to the mean of the gray values of our noisy background, was hardest to detect. Therefore, this contrast was the lower boundary of the staircase. Trial timing and stimulus duration remained the same as in the tactile threshold estimation process.

Following the threshold estimation in tactile and visual modalities, participants performed a short *unimodal training* in both conditions to verify thresholds calculated from the adaptive staircase procedure. The order of modalities was chosen randomly. Trial timing remained the same as in the habituation tasks. To keep performance at a comparable level, thresholds were adjusted if accuracy was below 75% or above 85% over five blocks. For the adjustment, the same step sizes as in the adaptive staircase were used.

#### Visuo-Tactile Matching

After the unimodal threshold estimation, participants conducted the visuotactile matching task. In this task, visual and tactile patterns were presented with synchronous onset and offset, and participants had to decide whether the patterns were congruent or incongruent. Participants responded with the left index (“congruent”) or middle finger (“incongruent”) *via* button press on a response box and again visual feedback (a green “+” or a red) was given in every trial. Trial timing was the same as in the unimodal recognition task (Figure 1C). Congruent and incongruent stimulus pairs were presented equally often. Participants started the visuotactile matching task at the stimulus intensity of the unimodal thresholds and performed a set of five consecutive blocks, consisting of eight trials. If participants did not reach an average accuracy between 75% and 85% correct within these five blocks, visual and tactile stimulus intensities were adjusted. Stimulus intensities in both modalities were either increased (accuracy <75%) or decreased (accuracy >85%) according to the steps of the respective unimodal adaptive staircase procedure.

After adjustment of stimulus intensities participants performed another set of five blocks. The experiment ended when participants reached a stable performance between 75–85% correct averaged over a set of five blocks (mean number of sets = 2.25, *SD* = 0.93).

### Statistical Analysis

Statistical analyses were performed using Matlab (Version 8.4.0.150421, MathWorks, Natick, MA, USA, 2014) and RStudio (Version 3.5.4, R Core Team, 2017).

To test for baseline group differences a multivariate analysis of variance (MANOVA) was performed by means of R’s *manova*() command to investigate the relationship between the values for sex, MDT, 2-point-discrimination, MMSE, DemTect as dependent variables and group (younger vs. older) as the independent variable. As group allocation was defined by participants’ age, age was not included in the model. For *post hoc* analysis, two-sample *t*-tests were performed and Benjamini-Yekutieli (BY) correction was applied to adjust for multiple comparisons (Benjamini and Yekutieli, 2001).

Distribution tests of the task performance data revealed that multiple estimates were not normally distributed in both groups and each of the tasks (habituation tasks, unimodal training, visuotactile matching). We, therefore, opted to use non-parametric testing with consequent correction for multiple comparisons. Two-tailed Wilcoxon signed-rank tests were used to compare task performance between and within groups and BY correction was applied to adjust for multiple comparisons. For all analyses, the adjusted *p* values are given.

In the habituation task, two-tailed Wilcoxon signed-rank tests were used to compare performances in visual and tactile pattern detection between groups. BY correction was applied to adjust for multiple comparisons. Two-tailed Wilcoxon signed-rank tests were used to compare accuracies and thresholds before and after the unimodal training and between the two groups. BY correction was applied to adjust for multiple comparisons. As in the course of the visuotactile matching task pin height and gray-intensity were adjusted evenly according to the steps of the adaptive staircases, changes in stimulus intensities were highly dependent. Two-tailed Wilcoxon signed-rank tests and BY correction were used to compare accuracies and stimulus intensities between the groups in the first and last set of five blocks of the visuotactile matching task. In addition, two-tailed Wilcoxon signed-rank tests and BY correction were performed to compare accuracies and stimulus intensities within the groups between the first and the last set of the visuotactile matching task. To evaluate detection performance for congruent and incongruent stimulus pairs, two-tailed Wilcoxon signed-rank tests were used to compare accuracies within and two-sampled *t*-tests to compare accuracies between groups. BY correction was used to adjust for multiple comparisons.

For all pairwise comparisons of task performance, effect sizes were calculated by dividing the standardized test statistic Z by the square root of the number of pairs (N).

## Results

### Baseline Data

The group comparison of baseline data obtained in the assessment prior to inclusion (Table 1) showed significant differences between the groups of younger and older participants (Pillai’s Trace = 0.43, *F* = 5.15, *df* = (1,38), *p* < 0.01). *Post hoc* comparison of the baseline data showed that DemTect scores (*p* < 0.001) differed significantly between groups. Importantly, the measurements revealed age-appropriate, not pathological results in the older group.

**Table 1:** Baseline data of the groups.

Metrics	Younger group (*n* = 20)	Older group (*n* = 20)
DemTect	17.8 (± 0.6)*	15.9 (± 1.5)*
MMSE	29.7 (± 0.6)	29.6 (± 0.6)
2-Point (mm)	2.1 (± 0.2)	2.2 (± 0.4)
MDT (mN)	0.28 (± 0.1)	0.57 (± 0.5)

Mean values are shown ± standard deviation. Based on a significant main effect of the factor group (younger group vs. older group), post hoc tests were conducted. *Indicate a significant difference between younger and older participants, *p*-value ≤ 0.01.

### Habituation Tasks

The analysis of performance in the habituation tasks revealed significant differences between groups in the unimodal tactile task. The younger participants (95.70 ± 5.10%) performed significantly better compared to the older group (82.47 ± 10.44%), (*Z* = 3.95, *p* < 0.001, *r* = 0.88). In the visual task, response accuracies did not differ between the groups (Younger group: 98.12 ± 2.94%/Older group 98.12 ± 2.94%; *Z* = 0, *p* = 1).

### Threshold Estimation and Unimodal Training

**Figure 2:**
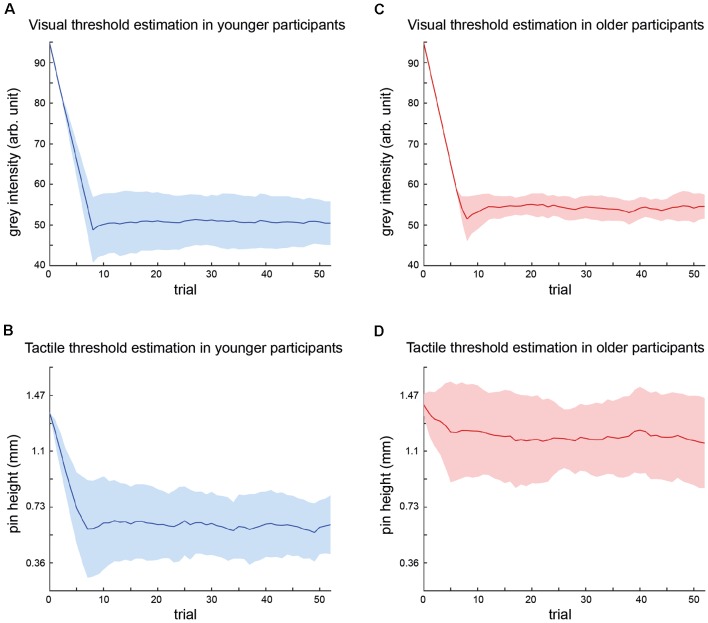
Summary of threshold estimations for visual and tactile stimulus intensities. Graphs depict the mean stimulus intensity (y-axis) per trial (x-axis) during the course of the adaptive staircase over all participants (younger group = blue; older group = red) with standard deviations (SDs; colored areas). The number of trials equals trials in shortest threshold estimation procedure, i.e., trials common to all participants. **(A)** Visual threshold estimation in younger participants. **(B)** Tactile threshold estimation in younger participants. **(C)** Visual threshold estimation in older participants. **(D)** Tactile threshold estimation in older participants.

The results of the threshold estimation are displayed in Figure 2.

Visual threshold estimation in the younger group resulted in a mean gray intensity of 49.2 ± 1.1. The mean adaptive staircase for tactile threshold estimation in the younger group showed a course similar to the visual condition and resulted in a mean threshold, i.e., pin height of 0.60 ± 0.17 mm.

Visual threshold estimation in the older group resulted in a mean gray intensity of 53.9 ± 2.5. The mean adaptive staircase for tactile threshold estimation in the older group showed only a small downward trend, indicating that the tactile threshold in the older group was close to maximum stimulus intensity. The mean tactile threshold in the older group was 1.13 ± 0.28 mm.

To ensure the validity of the estimated thresholds, the unimodal training was performed. Within the groups, there was no significant change of visual or tactile thresholds in the course of the training (older group: gray intensity *Z* = 1.27, *p* = 0.74; pin height *Z* = −1.80, *p* = 0.39/younger group: gray intensity *Z* = 0.51, *p* = 1; pin height *Z* = 1.55, *p* = 0.52), indicating a reliable threshold estimation. Across groups, there was no difference in detection accuracy (visual *Z* = 0.71, *p* = 1; tactile *Z* = 2.32, *p* = 0.15), but as expected in gray intensity (*Z* = −7.36, *p* < 0.001, *r* = −1.65) and pin height (*Z* = −6.75, *p* < 0.001, *r* = −1.51).

### Visuo-Tactile Matching

The mean accuracies, tactile (pin heights) and visual (gray intensities) stimulus intensities of the first and last set of five blocks of the visuotactile matching task were compared within and between groups (Table 2).

**Table 2:** First and last set of the visuotactile matching task.

	Younger group (*n* = 20)	Older group (*n* = 20)
First set of matching task	
Accuracy (%)	78.31 (± 9.09)*	66.20 (± 9.31)*^#^
Pin height (mm)	0.58 (± 0.17)*	1.14 (± 0.28)*^#^
Gray intensity	49 (± 1.38)*	53.65 (± 2.70)*^#^
Last set of matching task	
Accuracy (%)	79.50 (± 5.94)	77.28 (± 6.00)^#^
Pin height (mm)	0.57 (± 0.17)*	1.24 (± 0.27)*^#^
Gray intensity	48.9 (± 1.59)*	56.2 (± 2.69)*^#^

Mean values are shown ± standard deviation for accuracy, gray intensity and pin height in the first and last set of the task, sorted by group. *Indicate a significant difference between younger and older participants, all p-values ≤ 0.001; ^#^indicate a significant difference within the older group, all p-values ≤ 0.01.

In the first set, older participants performed significantly worse compared to the younger participants (*Z* = 3.63, *p* < 0.01, *r* = 0.81) despite their significantly higher unimodal stimulus intensities (gray intensity *Z* = −5.14, *p* < 0.001, *r* = −1.15; pin height *Z* = −4.72, *p* < 0.001, *r* = −1.06). To reach a performance of around 80% correct responses in the older group, visual and tactile intensities had to be further increased significantly according to the steps of the adaptive staircase (gray intensity *Z* = −3.83, *p* = 0.001, *r* = −0.86; pin height *Z* = −3.82, *p* = 0.001, *r* = −0.85). With this adjustment of stimulus intensity task performance was significantly improved (*Z* = 3.17, *p* < 0.01, *r* = −0.71) and there was no longer a significant difference in accuracy between the younger and the older group (*Z* = 1.13, *p* = 0.95). Within the younger group, there was no difference between the first and the last set in accuracy (*Z* = −0.20, *p* = 1), gray intensity (*Z* = 0.70, *p* = 1) and pin height (*Z* = 2.19, *p* = 0.12).

### Congruent vs. Incongruent Stimulus Pairs

To further explore the differences in performance in the visuotactile matching task, we analyzed detection accuracy for congruent and incongruent stimulus pairs separately.

**Figure 3:**
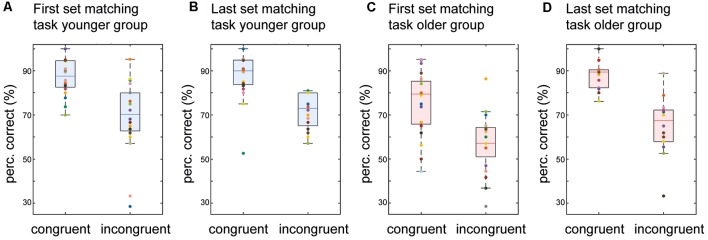
Detection accuracy of congruent vs. incongruent stimulus pairs. Boxplots of detection accuracy for congruent vs. incongruent stimulus pairs for the two groups in the first and the last set of the matching task. The boxes range from the first to the third quartile of the distribution, the line across the boxes indicates the median. The endpoints of the “whiskers” represent the lowest and largest data points excluding outliers. The colored dots represent individual participants. **(A)** Performance for the first set of the matching task in younger participants. **(B)** Same as **(A)** but for the last set of the matching task in younger participants. **(C)** Performance for the first set of the matching task in older participants. **(D)** Same as **(C)** but for the last set of the matching task in older participants.

Both age groups exhibited strong congruency effects with the detection rate for congruent patterns being significantly higher than for incongruent pairs over all the matching blocks (older group: congruent 82.12%/incongruent 62.21%, *Z* = 3.70, *p* < 0.01, *r* = 0.83; younger group: congruent 87.34%/incongruent 70.33%, *Z* = 3.37, *p* < 0.01, *r* = 0.76). To evaluate the changes in performance with adjustment of stimulus intensities, we analyzed performance in the first and the last set of the visuotactile matching task separately. While overall detection accuracy was significantly lower in the older group in the first set of the visuotactile matching task (“Visuo-tactile Matching” section), the difference in detection accuracy for congruent vs. incongruent stimulus pairs was the same (18%) for both age groups (older group: 75.48% vs. 57.11%, *Z* = 3.20, *p* < 0.01, *r* = 0.72/younger group: 87.05% vs. 69.30%, *Z* = 3.49, *p* < 0.01, *r* = 0.78; no difference between groups in percentage difference, *Z* = −0.29, *p* = 1; see Figure 3).

Adjustment of stimulus intensities increased the mean accuracy of pattern detection in the last set of the visuotactile matching task in the older group (“Visuo-tactile Matching” section). Analyses for congruent vs. incongruent stimulus pairs showed that this effect was driven by better detection of congruent patterns. With increased stimulus intensity, there was a significant increase in the detection of congruent stimulus pairs (*Z* = −2.92, *p =* 0.01, *r* = −0.65), while the detection of incongruent pairs was not significantly enhanced (*Z* = −1.79, *p* = 0.23). Due to this asymmetric benefit, the congruency effect increased to 23% (88.12% vs. 65.47%, *Z* = 3.66, *p* < 0.01, *r* = 0.82; see Figure 3D) in the last set of the matching task in the older group.

## Discussion

This study aimed to investigate performance differences in visuotactile pattern matching between younger and healthy older adults. The data show that older participants performed worse in unimodal pattern recognition and had higher unimodal detection thresholds. The main finding was that in the crossmodal condition older participants showed higher thresholds compared to the unimodal condition, while younger participants showed a stable performance. However, the performance of older participants could be enhanced by further increasing stimulus intensity. This effect was driven by higher detection rates for congruent stimulus pairs, while the detection of incongruent pairs was not significantly enhanced. These findings indicate that congruency effects can attenuate the difficulties of older adults in complex crossmodal tasks such as visuotactile pattern matching.

Confirming our first hypothesis, older participants showed significantly higher thresholds for unimodal tactile and visual pattern recognition than the younger. This is in line with previous findings and most likely caused by age-related decline of sensory organs (Mahoney et al., 2011; Mozolic et al., 2012). The data indicate that healthy older adults are able to perform at a comparable level of accuracy but require higher stimulus intensities (Humes et al., 2009). Contrary to our second hypothesis, we did not find evidence for enhanced crossmodal interactions in the older participants. The data showed that in the older group the stimulus intensities required for successful crossmodal pattern matching were significantly higher compared to the unimodal conditions. Younger participants performed significantly better compared to the older participants in the crossmodal task at the individually defined perception thresholds. Required stimulus intensities in the younger group did not differ between the unimodal and the crossmodal condition. However, even in the complex visuotactile matching task, older participants were able to reach the same level of performance as in the unimodal detection task. This enhancement of performance with increased stimulus intensities was driven by better detection of congruent stimulus pairs, while the detection of incongruent stimuli did not improve, resulting in a numerically stronger congruency effect (23%) than in younger adults (18%).

As stimulus intensities were individually adjusted to achieve comparable unimodal task difficulty for the younger and the older group, our data suggest that poor performance of older participants in the crossmodal task was not related to the processing of sensory stimuli, but a decline of mechanisms relevant for crossmodal pattern matching. In contrast to the unimodal condition as well as other classical multisensory integration tasks, stimuli in the visuotactile matching task arise from two different locations. Participants have to pay attention to visual and tactile stimulation concurrently and identify patterns separately in both modalities before comparing them. This might be seen as a worst-case scenario for crossmodal interactions as one could argue that the integration of the stimuli is not facilitated in a bottom-up manner but requires divided attention to both stimuli. As top-down mechanisms such as attention tend to decline with age and lead to processing difficulties of incoming stimuli (Guerreiro et al., 2014), this might be a major reason for the poor performance of older participants in the crossmodal condition. Hein and Schubert (2004) suggested that impaired top-down control in older adults leads to difficulties in scheduling attention across multiple input channels during dual-task situations (Poliakoff et al., 2006b). This might also apply to the visuotactile matching task. In line with our results, earlier studies indicated that older adults do not benefit from crossmodal stimulation in very complex tasks involving sensory as well as higher-order cognitive processes (Sommers et al., 2005; Mozolic et al., 2012; Freiherr et al., 2013). Taken together, we did not find evidence for enhanced crossmodal interactions in older adults. The mechanisms that are thought to lead to enhanced crossmodal integration in older adults, such as the increase of baseline noise (Mozolic et al., 2012), general cognitive slowing (Setti et al., 2014) or inverse effectiveness associated with sensory deficits (Freiherr et al., 2013) do not seem to apply in our experimental setting requiring divided attention to identify and match crossmodally presented patterns.

However, our data show that with increasing stimulus intensities, healthy older adults were able to improve performance in the visuotactile matching task. Interestingly, performance increased only for congruent stimulus pairs. The beneficial effects of congruent crossmodal stimulation have been described before. Initially, it was shown that crossmodal stimulation delivering corresponding cues to two modalities speeds up reaction times compared to unimodal stimulation alone (Miller, 1982, 1986). This so-called “redundant signal effect” has also been shown to apply for older adults and is thought to counteract age-related unimodal shortcomings (Laurienti et al., 2006; Mahoney et al., 2011). Similar effects have been shown in younger adults for crossmodal congruent vs. incongruent information perceived through various modalities (e.g., Spence et al., 2008; Göschl et al., 2014, 2015). There is extensive literature on this so-called “crossmodal congruency effect” derived from the “crossmodal congruency task” (e.g., Spence et al., 2004, 2008; Poliakoff et al., 2006b). In the original visuotactile version of the crossmodal congruency task participants have to make speeded elevation discrimination responses to vibrotactile stimuli while trying to ignore simultaneously presented visual stimuli. In this task, congruent tactile and visual patterns lead to shorter reaction times and fewer errors compared to the incongruent condition, i.e., the crossmodal concurrency effect (Spence et al., 2008). It has been shown that this crossmodal congruency effect is relatively insensitive to top-down factors such as spatial attention (Spence et al., 2004; Shore and Simic, 2005). The authors suggest that this indicates an automaticity of the neural processes underlying the effect. Our data show crossmodal congruency effects for congruent vs. incongruent stimulation in younger and older participants. At the individual unimodal perception thresholds, congruency effects in the crossmodal task are similar in size in the younger and the older group. When stimulus levels are adjusted, better performance for congruent but not incongruent pairs drives the improved results in older participants in the visuotactile matching task. In line with the interpretation of the crossmodal congruency effect above, congruency of visuotactile patterns as a bottom-up stimulus property seems to attenuate the deficits of older adults in crossmodal pattern matching.

Another interesting approach is to view the current results in the scope of crossmodal correspondence (for review, see Spence, 2011; Spence and Deroy, 2013). The term crossmodal correspondences refer to our brain’s tendency to systematically associate certain features or dimensions of stimuli across different modalities (Spence and Deroy, 2013). The literature on crossmodal correspondences systematically reports advantages for stimuli that are crossmodally corresponding within the context of concurrent stimulation. Crossmodal correspondence has been shown to affect response speed as well as working memory performance (e.g., Brunetti et al., 2017, 2018). Another way to look at the visuotactile matching task is a comparison between geometrically corresponding (congruent) vs. not-corresponding (incongruent) crossmodal visuotactile stimuli. Therefore, even though not arising from the same object crossmodal correspondence of geometric patterns might facilitate cross-modal interactions and improve performance compared to the incongruent condition.

In summary, older participants performed worse in a complex visuotactile matching task at the individual unimodal perception thresholds. We do not find behavioral evidence for an enhancement of crossmodal interactions in the older compared to the younger group. Our data suggest that a decline of top-down mechanisms such as attention might decrease performance in visuotactile pattern matching. However, even in this complex task older participants were able to perform at a comparable level with younger adults when higher stimulus intensities were offered. The relative improvement in performance after this adjustment of stimulus intensities was driven by better detection of congruent stimulus pairs.

These findings might have implications for future applications of crossmodal tasks and scenarios. Paying attention to more than one modality and basing one’s decision on a wider range of cues has been suggested to compensate for impaired unisensory processing (Hairston et al., 2003). Following this idea, Laurienti et al. (2006) suggested the use of crossmodal everyday life gadgets and multisensory training strategies for older adults. In the light of our results, one has to consider that the previously observed benefit of crossmodal integration in older adults might not necessarily be driven by the crossmodal nature of the task but, rather, by the congruency of the stimulus materials (Laurienti et al., 2006; Peiffer et al., 2007). Therefore, the current results might add certain limitations to the idea of crossmodal integration as a compensation mechanism for age-related impairments. These limitations include the type and familiarity of stimuli and the cognitive demands of a task. Complex cognitive tasks seem to lower the older adults’ capacity to compensate impairments. In this context, the observed benefit of congruent stimulus material might be exploited in future studies and practical applications. To use the effects of crossmodal interactions in everyday life, congruent information with high stimulus intensities should be delivered through the target modalities. Given the nature of our results, this might hold true not only for tasks concerned with spatial patterns. Frings and Spence (2010) show crossmodal congruency effects in a task requiring participants to identify temporal patterns (i.e., simple rhythms) presented simultaneously to different modalities. As such a task requires comparable top-down mechanisms as compared to the visuotactile matching task, one might speculate that results in an older group would resemble the results of the current study. Finally, one might expect that similar results could be obtained if the matching of complex patterns occurred on a temporal scale within one modality alone, again showing the beneficial effects of congruent stimuli. As one of the most important endeavors in aging neuroscience is to identify means to support older adults to maintain mental health and independent living, crossmodal congruency effects might be one asset to help older adults master cognitively demanding tasks or to cope with complex scenarios. Crossmodal congruency effects might also be used to develop strategies for the care of disabled older adults as, for example, in neurological rehabilitation.

There are some limitations to the current work. In the data presented here, variance in the older participants’ performance was larger compared to the younger group. Heterogeneity in older adults is likely to occur with respect to sensory and cognitive impairments. Moreover, highly relevant behavioral and physiological changes not only occur from young to old, but also in higher age (Poliakoff et al., 2006a). Considering these aspects, other studies divided participants into young, young-old and old-old. This approach offers the advantage of a more detailed view of the evolution of age-related changes and differences within the older population. Future studies investigating the effects of crossmodal interactions in older adults might consider recruiting more than two groups.

## Data Availability Statement

The datasets generated for this study are available on request to the corresponding author.

## Ethics Statement

The studies involving human participants were reviewed and approved by Local ethics committee of the Medical Association of Hamburg (PV5085). The patients/participants provided their written informed consent to participate in this study.

## Author Contributions

FLH: study design, data acquisition, data analyses, interpretation, and preparation of the manuscript. CH: data acquisition, data analyses, interpretation, and preparation of the manuscript. LK and FG: study design, interpretation, and revision of the manuscript. AE: study idea, interpretation, and revision of the manuscript. FCH: study idea and revision of manuscript. GX: study idea, interpretation, and revision of the manuscript. CG: study idea, study design, interpretation, and revision of the manuscript.

## Conflict of Interest

The authors declare that the research was conducted in the absence of any commercial or financial relationships that could be construed as a potential conflict of interest.
